# *Diplotaxis tenuifolia* (L.) DC. Yield and Quality as Influenced by Cropping Season, Protein Hydrolysates, and *Trichoderma* Applications

**DOI:** 10.3390/plants9060697

**Published:** 2020-05-30

**Authors:** Gianluca Caruso, Christophe El-Nakhel, Youssef Rouphael, Ernesto Comite, Nadia Lombardi, Antonio Cuciniello, Sheridan Lois Woo

**Affiliations:** 1 Department of Agricultural Sciences, University of Naples Federico II, 80055 Portici, Italy; christophe.elnakhel@unina.it (C.E.-N.); youssef.rouphael@unina.it (Y.R.); ernesto.comite@unina.it (E.C.); nadia.lombardi@unina.it (N.L.); 2 Council for Agricultural Research and Economics (CREA)—Research Center for Cereal and Industrial Crops, 81100 Caserta, Italy; antonio.cuciniello@crea.gov.it; 3 Department of Pharmacy, University of Naples Federico II, 80131 Naples, Italy; woo@unina.it; 4 Task Force on Microbiome Studies, University of Naples Federico II, 80055 Portici, Italy; 5 National Research Council, Institute for Sustainable Plant Protection, 80055 Portici, Italy

**Keywords:** *Trichoderma harzianum* strain T22, synergistic biostimulants, perennial wall rocket, mineral content, organic acids, chlorophylls, antioxidant activity, total ascorbic acid

## Abstract

Increasing attention is being given to plant biostimulants as a sustainable farming practice aimed to enhance vegetable crop performance. This research was conducted on greenhouse-grown perennial wall rocket (*Diplotaxis tenuifolia* (L.) DC.), comparing three biostimulant treatments (legume-derived protein hydrolysates, *Trichoderma harzianum* T22, and protein hydrolysates + *Trichoderma harzianum* T22) plus an untreated control, in a factorial combination with three cropping seasons (autumn–winter, winter, winter–spring). Measurements were performed on leaf yield components, colorimetric indicators, mineral composition, bioactive compounds, and antioxidant activity. Leaf marketable yield and mean weight, as well as plant dry weight, showed the highest values in winter crop cycle. Biostimulant treatments resulted in 18.4% and 26.4% increase in leaf yield and number of leaves per rosette, respectively, compared to the untreated control. Protein hydrolysates led to the highest plant dry weight (+34.7% compared to the control). Soil plant analysis development (SPAD) index as well as NO_3_, PO_4_, SO_4_, and Ca contents were influenced more during the winter–spring season than the winter cropping season. The winter production season resulted in a 19.8% increase in the leaf lipophilic antioxidant activity, whereas the hydrophilic antioxidant activity was 34.9% higher during the winter–spring season. SPAD index was the highest with protein hydrolysates + *Trichoderma* applications, which also increased the colorimetric parameters compared to the untreated control. The treatment with protein hydrolysates + *Trichoderma* enhanced N, PO_4_, Mg, and Na contents, compared to both biostimulants applied singly and to the untreated control. Both biostimulants applied alone or the protein hydrolysates + *Trichoderma* combination led to the increase of the lipophilic and hydrophilic antioxidant activity, as well as ascorbic acid and chlorophyll b, compared to the untreated control. The present research revealed that protein hydrolysates and *Trichoderma* single applications, and even more their combination in the case of some nutrients content, represent an effective tool for enhancing the yield and the quality attributes of perennial wall rocket produced under the perspective of sustainable crop system.

## 1. Introduction

Perennial wall rocket (*Diplotaxis tenuifolia* (L.) DC.) is a leafy vegetable species that has been increasingly cultivated in the Mediterranean area in the past two decades, accounting for a surface area of 4000 ha of horticultural production in Italy [1]. The leaves are mainly used raw and in salads, and this product has found a worldwide diffusion in both the fresh and baby leaf oriented grocery markets. This produce is appreciated by the consumer not only for its pungent “peppery” taste, but also for its high beneficial nutritional and antioxidant components, which are noted functions in the prevention of cardiovascular and carcinogenic diseases [2].

*D. tenuifolia* is mostly cultivated in greenhouses and the crop cycles are usually managed from early autumn to spring or from spring to summer, depending on farming systems, growing area, and commercial demand [1,2]. On average, five to six crop cycles are possible, each with a duration of 20 to 100 days after planting or regrowth, according to the time frame. The longest period occurs in winter due to the lower temperatures and light intensity, which result in slower plant growth [1,2].

The current trends in agricultural production towards sustainable cropping systems, aimed to fulfill the food demands of an increasing world population or meet consumer expectations for functional and healthy foods, have elicited researchers to identify suitable farming strategies and solutions. One possibility is the use of biostimulants [3], such as vegetal-derived protein hydrolysates (PHs), proven to enhance plant root and biomass development, even in unfavorable soils and environmental conditions [4], increase yield, and limit nitrate accumulation [5]. Some protein hydrolysates have an auxin-like action due to the presence of specific peptides, which serve as signaling molecules activating the processes of plant auxin biosynthesis that stimulate plant growth, i.e., tomato rhizogenesis [4]. However, the benefits stemming from biostimulant applications depend upon the formulation, dose, time of application, plant species, developmental stage, and environmental conditions [6]. Moreover, PHs were proven to stimulate plant primary metabolism [7], as reported in the case of the vegetal-derived PHs in improving nitrogen assimilation in maize plantlets [8]. Inhibiting effects were also noted on root uptake and the reduction activity of nitrate, as well as on amino acids such as glutamine and arginine that act as signaling molecules in regulating NO_3_^−^ absorption [9,10].

Apone et al. [11] reported that the positive effects of protein hydrolysates and amino acids in eliciting plant secondary metabolism and enhancing the tolerance to abiotic stresses, depended upon the involvement of antiaging specific genes. Other researchers [12] observed an increase of secondary metabolites when the PHs were applied to lettuce grown in saline conditions, which improved the plant tolerance to salinity by adjusting the signals related to the defense pathway.

Furthermore, beneficial fungi belonging to the genus *Trichoderma* are noted for their biostimulant effects on plants [13]. In the interactions with the plant, *Trichoderma* communicates with the roots through a chemical crosstalk involving the release of auxins, small peptides, volatile substances, and other active metabolites, which results in the enhancement of root ramification, nutrient absorption, plant growth, and production [14]. However, the effectiveness of the fungus–plant relationship depends on microbial strain as well as on the environment and farming system [15,16].

The present research aimed to assess the potential effect of two different categories of biostimulants, a vegetal protein hydrolysate and a plant beneficial microbe, on crop cycle length, leaf yield, and quality performance of perennial wall rocket grown during three cropping seasons from autumn to spring.

## 2. Results and Discussion

### 2.1. Trichoderma Harzianum Strain T22 and Protein Hydrolysate Compatibility

**Figure 1:**
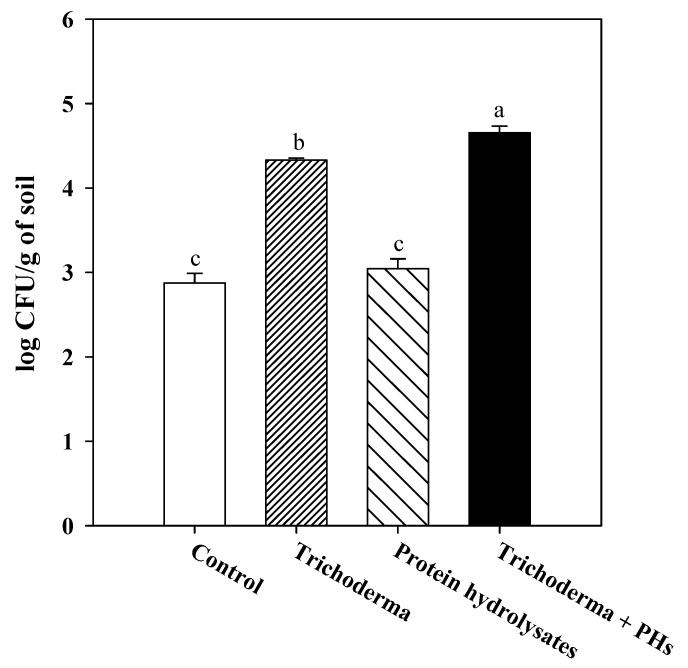
The number of *Trichoderma* colony forming units (CFU) at the end of the field experiment in soil samples collected in the wall rocket rhizosphere after treatments with *Trichoderma* and the protein hydrolysates (PH) biostimulant, singly and in combination. Values are significant at *p* ≤ 0.001, and values followed by different letters are significantly different according to Fisher test at *p* ≤ 0.05.

No significant differences were noted in the number of *Trichoderma* colony forming units (CFU) when cultured in the presence and absence of the PH biostimulant in the in vitro experiments (data not shown). The CFU of *Trichoderma* in soil samples collected from the rhizosphere of wall rocket at the end of the experiment demonstrated a significantly greater abundance in the treatments where the beneficial microbe was applied to the plants in comparison to those of the untreated control and the protein hydrolysates alone (*p* < 0.001; Figure 1). No significant differences were noted between the control and PH, indicating the baseline concentration of indigenous *Trichoderma* spp. in the soil. Furthermore, the combination of the two biostimulants greatly improved the development of the *Trichoderma* population in the presence of PH that was greater than the application of *Trichoderma* alone over time, suggesting a nutritive effect of the vegetal product that sustained the fungal colonization in the root zone.

### 2.2. Yield and Dry Weight

As reported in Table 1, the autumn–winter crop cycle was the longest and the winter–spring the shortest; crop duration was not significantly affected by the biostimulant applications.

The marketable yield components of wall rocket leaves were only evaluated in terms of total fresh weight (Table 1). The number of leaves per rosette and the mean leaf weight were the greatest during cultivation in the winter cropping season, when compared to the autumn–winter and winter–spring seasons.

Rocket yield increased with the biostimulants, showing an average yield 18.6% greater than the untreated control, but no significant differences were recorded among the three biostimulant (PH, *Trichoderma*, and their combination) treatments. Similarly, an average increase of 26.4% was noted in the number of leaves per rosette with the biostimulant applications, when compared to the control. There was a greater positive effect on mean leaf weight with *Trichoderma* inoculation, whereas the plant dry weight was enhanced by all biostimulant treatments with the highest increase elicited by the protein hydrolysates (+34.7%).

Positive effects of protein hydrolysates applications on the fresh yield components of perennial wall rocket were similarly reported in different horticultural commodities, including *Diplotaxis tenuifolia* [3], as well as baby leaf lettuce and spinach [17] and Vesuvian Piennolo tomato PDO [18]. PHs biostimulants contain components of low molecular size, such as peptides and free amino acids that can be readily absorbed through the leaves and roots, thus able to regulate plant development processes, for example, by the promotion of endogenous phytohormone biosynthesis [19]. In this regard, Kulkarni et al. [20] detected a 175% increase in cytokinin content in spinach plants treated with biostimulants in comparison to the untreated control. Moreover, it was observed that plant-derived PHs applied to lettuce could also stimulate the development of indigenous beneficial populations in the soil of the rhizosphere, such as N_2_-fixing, P-solubilizing, and indoleacetic acid-producing bacteria, thus suggesting a microorganism-mediated biostimulant action on plant growth [17]. Furthermore, among the different components present in PHs, it was demonstrated that soluble peptides and amino acids could act as signaling molecules involved in N metabolism, a process that positively affects the plant growth and thus crop productivity [21].

The applications of legume-derived PHs were shown to improve macronutrient uptake and assimilation, which subsequently modulates the root architecture and enhances the microbial-mediated soil nutrient availability [17,22]. In this respect, the amino acid tryptophan was noted as the main active physiological precursor for the synthesis of indole-3-acetic acid (IAA), which has a crucial influence on root architecture development [23,24].

The means by which numerous *Trichoderma* spp. establish beneficial interactions with the plant are various and diverse depending upon the species strain as well as the host plant [13,14]. For example, *T. harzianum* strain T-203 was found to enhance pepper leaf length and area, and the dry plant weight of cucumber [25], due to its ability to survive and develop in the rhizosphere, its capacity to inhibit pathogens, or contrast the negative effects of their phytotoxins, thus acting as a biocontrol agent [26,27]. Some *Trichoderma* species strains, such as *T. harzianum* strain T22, produce secondary metabolites that have auxin-similar activity and the release of small peptides or volatile organic compounds in the rhizosphere, which can positively influence plant root and crown development, or stimulate the defense response [28,29,30]. The positive effect observed on soybean seedling growth was a consequence of the root development and the increased bioavailability of nutrients from the soil to the roots as promoted by *T. harzianum* strain T-soybean [31]. It was also noted that the applications of T-soybean increased significantly the soil enzyme activity, which in its turn improved the growth and activity of soil microbes in terms of number, diversity, and biomass [32]. The biological activity of the microorganisms subsequently enhanced the soil fertility, thus contributing as a mechanism of growth promotion. Soil conditions are affected by the activity of enzymes in the soil; compounds which are produced by the microbial community also serve as a biological indicator of soil fertility [33]. For example, it was observed that cucumber growth and yield were associated with enzyme activities in the soil where they were cultivated [34]; and the growth-promoting effect on soybean could be related to 1-aminocyclopropane-1-carboxylate deaminase (ACCd) and siderophore activity with the *Trichoderma* treatment, as well as to the ability of the fungus to positively influence nitrogen and nutrient availability. Bio-organic fertilizer applications enriched with *T. harzianum* strain SQR-T037, a combination with the chemical fertilizer used at 75% of the recommended rate, resulted in tomato plant growth and yields similar to those observed with the full 100% dose of the chemical fertilizer without inoculants [35].

Other authors indicated that depending upon the plant species and genotypes, or the strain of *Trichoderma* applied, the effects on plant growth can be highly variable [15]. Notably, Ousley et al. [36] showed that lettuce growth parameters were significantly ameliorated when inoculated with six strains of *Trichoderma*, whereas Baker [37] outlined a restricted increase in crop performance of radish and pea with the same strains.

In a previous investigation conducted in the field, it was noted that cotton productivity was increased up to 300% upon the application of *Trichoderma hamatum* or *Trichoderma koningii* [38]. Similarly, in tomato the increased plant growth and productivity could be associated to the improved root growth, in response to harzianolide, an auxin-like phytohormone, which is secreted by *T. harzianum* [39]. This effect on tomato was observed in plants with an increase in total root length and tips by 1.5- to 2.6-fold, thus enabling the developing roots to extend in a greater area/volume of soil to improve the uptake of bioavailable nutrients.

### 2.3. SPAD Index and Leaf Colorimetric Parameters

As no significant differences arose between the autumn–spring and winter cycles regarding all of the examined qualitative parameters, only the results relevant to the winter and winter–spring cycles are reported in Table 2.

The soil plant analysis development (SPAD) index, representative of the efficiency of chlorophyll biosynthesis and activity/function of the photosynthetic system, was significantly affected by the period of the cropping season, taking into account that rocket leaves showed the highest values in winter–spring season compared to the winter one (Table 2).

The leaves of biostimulant-treated rocket plants exhibited higher SPAD values than those of the control, in particular under the *Trichoderma* + protein hydrolysates treatment. This combined application with the beneficial fungus and the biostimulant also affected leaf colorimetric parameters, producing an increase of L*, a*, and b* parameters, compared to the untreated control.

The increased efficiency of chlorophyll synthesis is mainly associated to the enhanced N uptake efficiency that affects crop outcome [20,40,41,42,43]. In a previous study, it was observed that the foliar application of PHs biostimulants elicited the increases of leaf SPAD index as much as 38.9% in lettuce leaves [44] and 18.9% in spinach leaves compared to the untreated control [45]. Russell et al. [46] reported that biostimulants promote the opening of leaf stomata in pea, which provides peptides an easier entrance and penetration through these natural openings with the foliar application, rather than via membrane transporters following drench application [47]. Similarly, biostimulant-treated jute plants exhibited higher photosynthetic activity, SPAD index, and improved leaf mineral status by accumulating more K and Mg, but less Na, showing a better accumulation and translocation of assimilates to the photosynthetic sinks, which result in improved crop performance.

### 2.4. Dry Residue and Organic Acid Content

Leaf dry residue of perennial wall rocket was not affected by either cropping season or by biostimulant treatment (Table 3). The cropping season significantly affected organic acid content with a 25.8% increase of malate in the winter season compared to the winter–spring season, whereas tartrate, oxalate, and citrate demonstrated higher concentrations in winter–spring season by significant differences of 50.0%, 24.3% and 17.5%, respectively (Table 3).

Regarding the diverse biostimulants applied to wall rocket, no differences were noted in the organic acid content, except for the *Trichoderma* + protein hydrolysates combination, which enhanced the oxalate content by 9.3% compared to the untreated control. In a previous study [42], spinach leaf dry matter was not significantly affected by the PHs-based biostimulant, confirming our observation in rocket. In contrast, protein hydrolysates applications were found to significantly increase the organic acids such as malate, oxalate, citrate, and isocitrate in tomato fruits [18]. In this respect, it was observed that the metabolic pathways were altered, particularly the Krebs cycle and glycolysis in maize plants treated with PHs [19,48]. Moreover, Schiavon et al. [48] indicated that the activities of numerous enzymes involved in C metabolism, such as malate dehydrogenase, NAD-dependent glutamate dehydrogenase, isocitrate dehydrogenase, and citrate synthase were stimulated by PHs application.

### 2.5. Mineral Composition

As observed in Table 4, the content of NO_3_, PO_4_, SO_4_, and Ca in rocket leaves was significantly higher in plants produced in the winter–spring season compared to those cultivated in winter. Five of these minerals were also affected by the biostimulant treatments, with the exception of SO_4_ where no significant differences were noted with any of the biostimulants. In particular, the nitrate content was an average of 23.3% higher with the three biostimulant treatments than the untreated control, but no significant differences were noted among the treatments. The combined *Trichoderma* + protein hydrolysates application increased the total nitrogen content, producing the highest values among all biostimulants. In addition, this fungal–biostimulant combination positively affected the phosphate (+18.2%), potassium (+23.3%), calcium (+19.2%), magnesium (+27.6%), sodium (+39%), and nitrate (+30%) contents in rocket leaves compared to the untreated control. Notably, protein hydrolysates applied alone resulted in a decrease in chloride concentration (−19.7%) in comparison with the control.

In a previous study, protein hydrolysates-based biostimulants improved the nutritional status of fruits from greenhouse cultivated tomato [49] by increasing K and Mg content compared to the untreated control. In baby leaf spinach, Rouphael et al. [45] found that PHs foliar treatment produced a 36.4% and 25.0% increase in K and Mg contents, respectively, and a lower Na/K ratio (0.014 vs. 0.025).

In the present research, it is conceivable that the soluble peptides and key amino acids present in the protein hydrolysates formulation could stimulate the metabolic processes in perennial wall rocket, resulting in increased K and Mg content in leaves [18,49]. This could be due to better nutrient uptake, translocation, and accumulation attributed to the modified root architecture of the plants [4,50], or by the activity of macronutrient transporters in cell membranes by the stimulation of encoding genes [51,52].

Perennial wall rocket is a plant known to readily accumulate nitrate, whereby high concentrations of this compound in the produce could represent a potential health risk for the consumers [53]. In the present work, nitrate concentrations in the rocket leaves were always under the limits for the maximum acceptable levels of nitrate established by the EC Regulation N. 1258/2011: 6000 mg kg^−1^ of NO_3_ in produce from the 1 April to 30 September growing season and 7000 mg kg^−1^ of NO_3_ in rocket grown in the 1 October to 31 March period. Although some research [50,52] found the higher nitrate accumulation in melon and tomato biostimulants-treated plants, others reported that the application of commercial biostimulants reduced nitrate content in rocket [54] and other leafy vegetables (Swiss chard, spinach, pak choi, and lettuce) [55,56,57]. Other authors [52] reported in tomato cultivation that protein hydrolysates modulate the growth of plants and key genes expression involved in N assimilation, such as ammonia and nitrate transporters.

In the case of microbial biostimulants, Cai [58], Harman et al. [13], and Altomare et al. [59] reported that *Trichoderma* treatments rendered nutrients such as phosphorus more available in the rhizosphere, whereby a substantial portion of P present in the soil and used by the fungus becomes more easily exploitable by tomato plants after microbial lysis [59]. Phytase released by *T. harzianum* plays an important role in solubilizing organic P, i.e., phytate [60]. Moreover, the introduction of *Trichoderma* SQR-T037 improved soil available nutrients, which in turn was positively correlated with soil microbe abundance [61].

### 2.6. Antioxidant Compounds and Activity

In our experiment, the factor of cropping season significantly affected leaf antioxidant activities, but not the content of the antioxidant compounds (Table 5). Indeed, the leaves harvested in the winter season had a 19.8% higher lipophilic antioxidant activity than those of the winter–spring season, whereas the rocket produced in the winter–spring season showed a 34.9% higher hydrophilic antioxidant activity than the plants grown in the winter period.

The effects of the biostimulant treatments, specifically with *Trichoderma* and *Trichoderma* + protein hydrolysates, elicited a 33.6% increase in the lipophilic antioxidant activity compared to the untreated control. The hydrophilic antioxidant activity was 18.8% greater on average than the control with the biostimulant applications.

Total ascorbic acid concentration in wall rocket leaves increased by 23.3% under *Trichoderma* and *Trichoderma* + protein hydrolysates treatments. The biostimulants also led to chlorophyll b enhancement (14.9% on average), but did not significantly affect the other antioxidant components (Table 5).

The synthesis and accumulation of health-beneficial antioxidant compounds could be related to the enzymatic activity in processes involving phytochemical homeostasis and, as recorded in the present research, to the high tissue K and Mg content [6,62]. Consistently with our findings, the beneficial effects of organic nitrogen-based biostimulants on chlorophyll was also noted in baby lettuce [63]; most likely caused by amino acid abundance in PHs-treated plants, which augmented the chlorophyll pigment content and improved the net photosynthetic rate. Chlorophyll b is a component of the light-harvesting complex II (LHCII) and is crucial in dissipating excess energy [64]. The increase in chlorophyll could raise the quantum yield of O_2_ evolution and the photosynthetic capacity, therefore reducing the sensitivity to photoinhibition [65].

Results obtained from the present investigation were in agreement with findings from previous studies that demonstrated that protein hydrolysates boosted ascorbic acid content and antioxidant activity in fruits of greenhouse pepper [6] and tomato [49,62]. The latter positive effects could be assigned to the activation of the key enzymes involved in cell antioxidant homeostasis, whereas the increase in nutrient assimilation recorded in our trial did not presumably lead to the enhanced synthesis of amino acids such as phenylalanine and tyrosine, which are precursors of polyphenols [66,67,68,69].

In contrast to Kulkarni et al. [20], who indicated that the beneficial effect of applying biostimulants to spinach was noted in the increase of phenylalanine ammonia lyase, a prominent enzyme [70] having an important role in biosynthesis of phenolic acids, in the present study no change was observed in the phenol content due to treatments with the examined biostimulants.

## 3. Materials and Methods

### 3.1. Experimental Location and Conditions

The experiment was performed on *Diplotaxis tenuifolia* (L.) D.C. cultivar Nature (perennial wall rocket), a common cultivar cultivated in the Campania region, at the Department of Agricultural Sciences, University of Naples Federico II, Portici, Southern Italy, in the 2019–2020 seasons. The crop was grown in an unheated greenhouse, covered with a thermal polyethylene film, in a sandy loam soil (76% sand, 17% silt, 7% clay), with a pH of 6.9 and an electrical conductivity of 512 mS cm^−1^. During the cropping seasons, mean monthly temperatures inside the greenhouse were 10 °C in November, 6.7 °C in December, 3.1 °C in January, 6.9 °C in February, and 12.2 °C in March.

The one month old seedlings were transplanted on 2 November 2019, with an intra- and inter-row plant spacing of 20 cm. Each plot bed contained 80 rosettes (alveoli), for a plant density of 14.3 rosettes m^−2^, with 80 cm spacing between plots.

Crop management was performed using sustainable management practices, including an organic preplant fertilization with N, P_2_O_5_, and K_2_O (at a rate of 38, 10, and 30 kg ha^−1^, respectively), application of a 15 µm thick MaterBi biodegradable black mulching, and control of fungal diseases and pests when necessary, with copper oxychloride and azadirachtin treatments, respectively. During the three growing seasons, drip irrigation was activated when the soil available water at 10 cm depth dropped to 80%, and N, P_2_O_5_, and K_2_O were supplied by fertigations in each crop cycle at a dose of 112, 30, and 90 kg ha^−1^, respectively.

Mature leaves of rocket, at a stage ready for commercialization, were harvested by cutting 12 to 15 cm lengths of the leaves at a 3 to 5 cm level above the soil surface, in order to leave the vegetative apex undamaged, thus permitting more efficient regrowth [71]. Harvests were conducted on 10 January, 6 March, and 4 April 2019.

### 3.2. Experimental Protocol and Treatments

*Trichoderma harzanium* strain T22 was the commercial biofungicide Trianum-P, kindly provided by Koppert Biological Systems, Rotterdam, the Netherlands. The vegetal-derived PHs biostimulant Trainer^®^, provided by Italpollina S.p.A., Rivoli Veronese, Italy, is obtained by an enzymatic hydrolysis of legume proteins of vegetal origins, and is rich in free amino acids and soluble peptides. An aminogram of the product is described in Colla et al. [49].

The experimental design was a factorial combination based upon three cropping seasons (autumn–winter, winter, winter–spring) and three biostimulant treatments (*Trichoderma*, protein hydrolysates, *Trichoderma* + protein hydrolysates), plus an untreated control. A randomized complete block design was organized with the treatment distribution in the greenhouse, replicated three times. Each treatment plot measured 100 cm wide × 160 cm long bed, but only the central row plants were used for sampling the yield and quality determinations, to avoid a border effect.

The inoculations with *Trichoderma harzanium* strain T22 were performed just after transplant and at the middle of each cropping season, by manually irrigating 25 mL of a *Trichoderma* spore suspension (inoculum concentration of 1 × 10^7^ spores·mL^−1^) per plant. The plants were uniformly sprayed with the commercial plant biostimulant, at a dose indicated in previous published papers of 3 mL product L^−1^ water [49,62]. Three foliar spray applications were conducted with the PHs biostimulant in each cropping season, starting when the rocket leaves reached 6 cm in length, then the treatments were repeated at weekly intervals.

### 3.3. Sampling and Yield Assessment

At each time of harvest, the perennial wall rocket fresh yield was determined for each treatment plot. From each plot, twelve rosettes (alveoli) were randomly selected for the cuttings and used to determine the number of leaves and the mean fresh weight at harvest. At the end of each cropping season, dry weight was measured after placing all collected samples in a forced air oven at 70 °C for 72 h, then the dry weight of the leaves was calculated and expressed as a percentage of the fresh weight.

The dried plant material was ground to a powder in a grinder, then a 500 mg sample was used for the extraction and determination of the mineral, nitrate, and organic acids content. Fresh leaves from 12 plants randomly sampled from each treatment plot were collected and stored at −80 °C until extractions and analyses were performed on the antioxidant compounds (total phenols and total ascorbic acid), antioxidant activity, and pigments (chlorophyll a and b and carotenoids).

### 3.4. SPAD Index and Colorimetric Components

At the time of harvest, but before cutting the leaves, the soil plant analysis development (SPAD) index determination was done on twenty wall rocket leaves per replicate, using a Konica Minolta chlorophyll meter (model SPAD-502, Tokyo, Japan). At the same time, the leaf color parameters L *, a *, and b * were measured on 10 leaves per replicate by the means of a Minolta CR-300 Chroma Meter (Minolta Camera Co. Ltd, Osaka, Japan) [71].

### 3.5. Analysis of Total Nitrogen, Nitrate, Mineral Composition, and Organic Acids

Ground plant material (250 mg) was analyzed by ion chromatography (ICS-3000, Dionex, Sunnyvale, CA, USA) to determine the mineral content according to the method proposed by Rouphael et al. [62]. Potassium, calcium, magnesium, phosphorus, sulfate, sodium, chloride, ammonium, malate, tartrate, oxalate, and isocitrate were expressed as g kg^−1^ dry weight (dw). The organic acids were quantified by running samples through the same column as used for the anion analysis. For nitrate content, the value was determined by taking into consideration the dry matter percentage of each sample and expressing the final value as mg kg^−1^ fresh weight (fw). For the determination of the total nitrogen concentration, the Kjeldahl method was used and the results expressed as percentage of N in the plant sample [72].

### 3.6. Bioactive Compounds, Phostosynthetic Pigments, and Antioxidant Activities

Total ascorbic acid (TAA) and photosynthetic pigments (chlorophylls and carotenoids) were determined on fresh plant material, whereas total phenols, lipophilic antioxidant activity (LAA), and hydrophilic antioxidant activity (HAA) were analyzed in freeze-dried material. These latter compounds were determined based on the methods described by Kampfenkel et al. [73], Arnon [74] and Lichtenthaler [75], Singleton et al. [76], Re et al. [77], and Fogliano et al. [78], respectively. All compounds were analyzed using a spectrophotometer (Hach DR 2000, Hach Co., Loveland, CO, USA); TAA, total phenols, LAA, and HAA solution absorbances were measured at 525, 765, 734, and 505 nm, respectively, whereas the chlorophyll a, chlorophyll b and carotenoid solution absorbances were measured at 662, 647, and 470 nm, respectively.

### 3.7. Compatibility Tests Between T22 and PHs In Vitro and in the Field

Tests were performed in vitro to determine the compatibility of *Trichoderma* T22 with the PH biostimulant. The final concentrations of each biological product, used singly and in combination, were equivalent to the concentrations used in the field experiment: T22 at 1 × 10^7^ spores mL^−1^, and the PH at a 1:330 dilution in water. Serial dilutions were made with Ringer solution (Sigma–Aldrich, Milano, Italy), then 100 µL was inoculated to 90 mm Petri plates containing *Trichoderma* Selective Media Agar (TSM; HiMedia Pvt. Ltd., Mumbai, India) augmented with 0.1% (*v*/*v*) Igepal (Sigma–Aldrich, Milano, Italy) and incubated at 25 °C. After five days, the number of *Trichoderma* colony forming units was determined. Each dilution series was plated in three replicates, and the experiment was performed twice.

At the time of harvest, soil samples were collected from the root–soil zone of four wall rocket plants from each treatment plot using a modified protocol of Rouphael et al. [63], to determine the *Trichoderma* CFU in the rhizosphere. A 1% (*w*/*v*) soil suspension was prepared in Ringer solution containing 0.162 g of sodium pyrophosphate (Sigma) and placed in horizontal agitation for 30 min; serial dilutions were prepared, and 100 µL was inoculated to 90 mm Petri plates containing TSM supplemented with Igepal and 0.01% (*w*/*v*) chloramphenicol (Sigma–Aldrich, Milano, Italy), then incubated at 25 °C. After five days, the *Trichoderma* fungal colonies were counted, as determined by visual and morphological identification.

### 3.8. Statistics

A two-way ANOVA was conducted on the experimental data using the SPSS software version 21. A Duncan’s or Fisher Multiple Range Test was performed at *p* ≤ 0.05 to compare the treatments means. Before processing, the percentage data were subjected to angular transformation.

## 4. Conclusions

The present research carried out on greenhouse-grown perennial wall rocket indicated that although the winter crop cycle produced a higher yield, the production in the winter–spring season developed leaves with a better overall quality. In fact, the winter–spring plants were greener and displayed a higher efficiency of chlorophyll biosynthesis and photosynthetic system activity; they also attained higher values of tartrate, oxalate, citrate, phosphate, sulfate, calcium, and hydrophilic antioxidant activity. Moreover, the single applications of protein hydrolysates to the leaves and *Trichoderma* to the roots, or the combined application of PHs and *Trichoderma*, were effective tools for enhancing leaf marketable yield, colorimetric parameters, mineral composition, and antioxidant activity of the plants in the crop seasons examined. Overall, the results of the present research demonstrated that, compared to the untreated control, both the application of nonmicrobial or microbial-based biostimulants alone or in combination activated various physiological mechanisms in perennial wall rocket plants, which enhanced leaf production by 18.4%, leaf quality traits, and hydrophilic (+33.6%) and lipophilic (+24.1%) antioxidant activities. In addition, the perennial wall rocket treated with the combined *Trichoderma* + protein hydrolysates exhibited greener leaves and a higher content of total nitrogen, phosphorus, and magnesium in comparison to the single applications of each biostimulant, thus obtaining produce with an added health value for the consumer. In conclusion, rocket producers can benefit from the use of the examined biostimulant applications to improve greenhouse vegetable farming practices, to increase the yield, and to enhance the nutritive value of produce under the perspective of a sustainable cropping system.

## Figures and Tables

**Table 1:** Effect of cropping seasons and treatments with three biostimulants on the marketable production parameters of perennial wall rocket leaves and on plant dry weight.

Source of Variation	Crop Cycle	Marketable Leaves			Plant Dry Weight (g m^−2^)
	Duration (days)	Yield (t ha^−1^)	Number per Rosette	Mean Weight (g)	
Cropping season					
Autumn–winter	69 a	9.3 c	118.7 b	0.55 b	105.48 c
Winter	55 b	19.1 a	213.9 a	0.69 a	171.51 a
Winter–spring	29 c	15.6 b	202.1 a	0.55 b	140.33 b
Biostimulant treatment					
*Trichoderma*	51	15.2 a	183.1 a	0.63 a	137.8 b
Protein hydrolysates (PHs)	51	14.9 a	191.5 a	0.57 c	156.1 a
*Trichoderma* + PHs	50	15.7 a	189.5 a	0.61 ab	146.6 ab
Untreated control	52	12.9 b	148.8 b	0.59 b	115.9 c
	n.s.				

n.s.: not statistically significant. Within each column, values followed by different letters are significantly different according to Duncan’s test at *p* ≤ 0.05.

**Table 2:** Cropping season and biostimulant effect on perennial wall rocket SPAD (soil plant analysis development) index and leaf colorimetric parameters.

Source of Variation	SPAD Index	Leaf Colorimetric Parameters		
		L *	a *	b *
Cropping season				
Winter	36.4	39.1	−13.5	19.3
Winter–spring	46.8	40.6	−14.1	20.8
	*	n.s.	*	*
Biostimulant treatment				
*Trichoderma*	42.7 ab	39.9 ab	−13.7 b	20.1 ab
Protein hydrolysates (PHs)	41.2 b	39.6 ab	−13.8 b	20.0 ab
*Trichoderma* + PHs	43.5 a	41.2 a	−14.6 a	21.1 a
Untreated control	39.1 c	38.7 b	−13.2 b	19.1 b

n.s.: not statistically significant; * significant at *p* ≤ 0.05. Within each column, values followed by different letters are significantly different according to Duncan’s test at *p* ≤ 0.05.

**Table 3:** Effect of cropping season and biostimulant treatment on perennial wall rocket leaf dry residue and organic acid content.

Source of Variation	Dry Residue (%)	Malate (g kg^−1^ dw)	Tartrate (g kg^−1^ dw)	Oxalate (g kg^−1^ dw)	Citrate (g kg^−1^ dw)
Cropping season					
Winter	9.0	18.68	0.08	0.70	17.33
Winter–spring	8.8	14.85	0.12	0.87	20.36
	n.s.	*	*	*	*
Biostimulant treatment					
*Trichoderma*	8.9	17.65	0.10	0.79 ab	20.30
Protein hydrolysates (PHs)	9.0	16.95	0.11	0.78 ab	17.56
*Trichoderma* + PHs	9.0	16.47	0.11	0.82 a	19.63
Untreated control	8.8	15.99	0.09	0.75 b	17.91
	n.s.	n.s.	n.s.		n.s.

dw: dry weight; n.s.: not statistically significant; * significant at *p* ≤ 0.05. Within each column, values followed by different letters are significantly different according to Duncan’s test at *p* ≤ 0.05.

**Table 4:** The influence of cropping season and biostimulant applications on perennial wall rocket leaf mineral content.

Source of Variation	Nitrate (mg·kg^−1^ fw)	Total N (%)	PO_4_	K	SO_4_	Ca (g·kg^−1^ dw)	Mg	Na
Cropping season								
Winter	6237	5.03	7.43	51.02	25.37	26.70	3.52	3.24
Winter–spring	6790	5.02	8.76	49.22	29.90	29.27	3.50	3.43
	*	n.s.	*	n.s.	*	*	n.s.	n.s.
Biostimulant treatment								
*Trichoderma*	6634 a	4.96 c	8.22 b	53.84 a	28.63	28.48 ab	3.57 b	3.43 b
Protein hydrolysates	6916 a	5.11 b	8.19 b	48.77 b	27.61	27.30 ab	3.39 b	3.22 b
*Trichoderma* + PHs	7127 a	5.25 a	9.05 a	54.04 a	28.21	30.54 a	3.97 a	3.89 a
Untreated control	5487 b	4.78 d	7.66 c	43.83 c	27.28	25.63 b	3.11 c	2.80 c
					n.s.			

fw: fresh weight; dw: dry weight; n.s.: not statistically significant; * significant at *p* ≤ 0.05. Within each column, values followed by different letters are significantly different according to Duncan’s test at *p* ≤ 0.05.

**Table 5:** Cropping season and biostimulant treatment influence on perennial wall rocket antioxidant compounds and activity.

Source of Variation	Lipophilic Antioxidant Activity (mmol Trolox eq.100 g^−1^ fw)	Hydrophilic Antioxidant Activity (mmol Ascorbic Acid eq.100 g^−1^ fw)	Total Phenols (mg Gallic Acid eq.100 g^−1^ fw)	Total Ascorbic Acid (mg 100 g^−1^ fw)	Chlorophyll a (mg g^−1^ fw)	Chlorophyll b (mg g^−1^ fw)	Chlorophyll a + b (mg g^−1^ fw)	Carotenoids (mg g^−1^ fw)
Cropping season								
Winter	2.11	0.098	34.3	20.07	0.89	0.54	1.43	0.33
Winter–spring	1.80	0.129	33.8	20.33	0.85	0.50	1.35	0.34
	*	*	n.s.	n.s.	n.s.	n.s.	n.s.	n.s.
Biostimulant treatment								
*Trichoderma*	2.14 a	0.119 a	33.7	21.22 a	0.86	0.52 a	1.38	0.34
Protein hydrolysates	1.85 b	0.113 a	33.8	18.27 b	0.89	0.55 a	1.43	0.33
*Trichoderma* + PHs	2.17 a	0.125 a	36.5	23.28 a	0.87	0.55 a	1.42	0.34
Untreated control	1.64 c	0.099 b	32.5	18.04 b	0.85	0.47 b	1.32	0.34
			n.s.		n.s.		n.s.	n.s.

n.s.: not statistically significant; * significant at *p* ≤ 0.05. Within each column, values followed by different letters are significantly different according to Duncan’s test at *p* ≤ 0.05.
